# Residual Properties of Geopolymer Concrete for Post-Fire Evaluation of Structures

**DOI:** 10.3390/ma16176065

**Published:** 2023-09-04

**Authors:** Balamurali Kanagaraj, Nammalvar Anand, Diana Andrushia, Venkatesh Kodur

**Affiliations:** 1 Department of Civil Engineering, Karunya Institute of Technology and Sciences, Coimbatore 641114, India; balakmurali31@gmail.com (B.K.); nanand@karunya.edu (N.A.); 2 Department of Electronics and Communication Engineering, Karunya Institute of Technology and Sciences, Coimbatore 641114, India; diana@karunya.edu; 3 Department of Civil and Environmental Engineering, Michigan State University, East Lansing, MI 48824, USA

**Keywords:** geopolymer concrete, elevated temperature, strength, microstructure

## Abstract

The research focuses on effectively utilizing industrial by-products, namely fly ash (FA) and ground granulated blast furnace slag (GGBS), to develop sustainable construction materials that can help reduce carbon emissions in the construction industry. Geopolymer mix design using these by-products is identified as a potential solution. The study investigates the impact of different water to binder ratios (W/B) ranging from 0.4 to 0.6 on the residual properties, including compressive strength (CS), of geopolymer concrete (GPC), in accordance with Indian Standard for Alkali activated concrete. Lower W/B ratios were found to result in a more compact and less porous microstructure in the GPC. Additionally, the research explores the post-fire performance of GPC with varying grades (M10, M20, M30, & M40) and different W/B ratios, following the ISO 834 standard fire curve. It was observed that concrete samples exposed to elevated temperatures displayed a more porous microstructure. The mass loss of GPC with 0.4 W/B was found to be 2.3–5.9% and for 0.6 W/B ratio, the loss was found to be 3–6.5%, after exposing to 30-, 60-, 90-, and 120-min of heating. In the case of strength loss, for 0.4 W/B ratio, the loss was 36.81–77.09%, and for 0.6 W/B ratio the loss was 38.3–100%, after exposing to 30-, 60-, 90-, and 120-min of heating. Overall, the findings suggest that optimizing the W/B ratio in geopolymer concrete can enhance its compressive strength, as well as residual properties, and contribute to its suitability as a sustainable construction material. However, the response to elevated temperatures should also be considered to ensure its performance in fire scenarios.

## 1. Introduction

Due to its adaptability, durability, and performance, concrete is the most frequently employed construction materials worldwide. Nonetheless, the production of cement, which serves as concrete’s principal component, and it is responsible for a notable proportion of carbon emissions. The cement industry is a primary cause for 7% of global carbon dioxide emissions [1]. Additionally, cement production consumes huge energy and natural resources. Apart from environmental concerns, cement concrete also has several other drawbacks [2]. One of the major drawbacks is its susceptibility to cracking due to its low tensile strength. This can lead to the formation of micro-cracks, which can eventually cause structural deterioration [3]. To mitigate this issue, steel reinforcement is typically added to the concrete, which adds to the cost and complexity of construction [4].

Another drawback of cement concrete is its poor resistance to chemical attack. When exposed to acidic or alkaline environments, the concrete can deteriorate, leading to a reduction in strength and durability [5]. This can lead to the need for costly repairs or even complete replacement of the concrete elements. Moreover, the production of cement requires significant amounts of water, which can be a scarce resource in some areas. The curing process of cement concrete also requires a significant amount of time, which can delay construction schedules. Therefore, there is a need to explore alternative building materials that are more sustainable and have fewer drawbacks than cement concrete.

Geopolymers are an innovative class of materials that serve as an alternative to traditional cement-based materials like concrete [6]. They are formed through a chemical process that involves the reaction of aluminosilicate source materials (such as fly ash, slag, or clay) with alkaline activators (such as sodium or potassium hydroxide and silicate solutions). The development of geopolymers involves these steps: Source Materials Selection: Aluminosilicate-rich materials are chosen as the primary ingredients [7]. These can be industrial byproducts such as fly ash, silica fume or natural materials like clay. Alkaline Activation: The aluminosilicate materials are mixed with alkaline activators, usually in a liquid form. The activators provide the necessary alkalinity to initiate the chemical reaction [8]. Polymerization: The alkaline activators induce a complex polymerization reaction in the aluminosilicate materials. This reaction forms a three-dimensional network of linked chains, similar to the structure of traditional cement hydrates [9].

Geopolymers require a curing period to develop their strength and durability. This curing process can occur at room temperature or under controlled heat conditions [6]. Advantages of Geopolymers: Reduced Environmental Impact [10]: Geopolymers use industrial byproducts like fly ash and slag, which reduces the need for mining of raw materials and lowers the carbon footprint associated with traditional cement production. High Early Strength [7]: Geopolymers can achieve rapid strength development, often faster than traditional concrete. This can lead to quicker construction processes. Durability: Geopolymers exhibit excellent resistance to chemical attack, corrosion, and fire, making them suitable for challenging environments. Reduced CO_2_ Emissions [11]: Geopolymer production emits significantly fewer greenhouse gases compared to conventional cement production, contributing to sustainable construction practices. Versatility: Geopolymers can be tailored for specific applications and can potentially replace traditional concrete in various scenarios. By utilizing industrial byproducts, geopolymers help to reduce waste and promote resource efficiency [12]. While geopolymers offer numerous advantages, they also face challenges, such as variability in source materials, the need for careful mix design and curing conditions, and limited standardization compared to traditional concrete [13]. Additionally, there is ongoing research to optimize the production process, enhance durability, and expand the range of applications for geopolymers.

The production process of GPC involves the synthesis of a geopolymer binder that acts as a replacement for cement. The geopolymer binder is formed by activating the industrial by-products with an alkaline solution, typically a mixture of sodium hydroxide and sodium silicate [6]. The synthesis of geopolymer binders involves a complex chemical reaction between the industrial by-products and the alkaline solution. The reaction results in the formation of a three-dimensional network of aluminosilicate chains, which provides the binding strength to the GPC [10]. The geopolymerization process can be accelerated by increasing the temperature during curing, typically between 60–90 °C. The final product is a high-strength, durable, and sustainable construction material, that is GPC [7].

The process of making GPC involves several steps, including mix design optimization, mixing of ingredients, casting, and curing [14]. Mix design optimization involves the selection of appropriate aggregates and industrial by-products, alkaline activator solution, and the water-to-binder ratio [15]. The mix design is critical to achieve the desired mechanical properties of the GPC. Once the mix design is optimized, the ingredients are mixed together in a concrete mixer. The mixing process is critical to ensure uniform distribution of the geopolymer binder and other components [16]. The mixture is then cast into moulds, which can be of various shapes and sizes, depending on the application. The curing process involves keeping the GPC in a moist and warm environment, typically between 60–90 °C, for a specific period, typically 24–48 h [17].

However, despite of its many advantages, GPC also has several drawbacks that need to be addressed. One of the major drawbacks is the relatively higher cost of production compared to traditional cement concrete [18]. The cost of production is mainly due to the high cost of the alkaline activator solution for the geopolymerization process [19]. Another major drawback of GPC is its lower workability compared to traditional cement concrete [20]. GPC has a shorter working time, which can make it challenging to cast complex shapes or structures. In addition, the high viscosity of the geopolymer binder can lead to segregation and bleeding of the mixture, which can compromise the mechanical properties of the final product [18].

Furthermore, the long-term durability of GPC is still not fully understood, particularly in harsh environments such as seawater exposure and freeze-thaw cycles. Seawater exposure: GPC generally displays favorable characteristics when exposed to seawater due to its resistance to chemical attack, reduced permeability, and potential resistance to alkali-silica reaction. However, while it holds promise for marine applications, ongoing research and testing are necessary to refine mix designs to understand its behavior over time when subjected to the unique challenges posed by seawater environments [21]. Freeze-Thaw: GPC typically has a different pore structure compared to traditional concrete. The geopolymerization process can lead to a more refined pore network with fewer capillary pores. This reduced porosity can potentially result in lower water absorption and improved freeze-thaw resistance [6]. However, the specific performance under freeze-thaw conditions can vary based on the specific geopolymer mix design, curing conditions, and other factors [22]. Geopolymer binders generally exhibit good thermal stability compared to traditional cement-based binders. They can withstand higher temperatures without undergoing significant loss of strength or structural integrity. Strength retainment at elevated temperatures can vary based on factors such as mix design, curing conditions, and specific raw materials used. In many cases, geopolymer concrete maintains a higher proportion of its original strength compared to conventional concrete when subjected to heat [23]. Also, there is lack of fire resistance properties of GPC limits its widespread use in infrastructure projects. Lastly, the availability and quality of industrial by-products such as fly ash and slag, which are the key components of GPC, can vary significantly depending on the location and market demand [24]. This can limit the scalability and consistency of GPC production. Therefore, addressing these drawbacks is crucial for the widespread adoption of GPC as a sustainable alternative to traditional cement concrete. Further research and development are needed to improve the workability, long-term durability, and scalability of GPC production.

Most of the past studies deal with the concrete subjecting to random temperature exposure. This means that in previous studies, researchers subjected concrete samples to varying and unspecified temperature conditions, possibly simulating fire or heat exposure, but without adhering to a specific standardized guideline. The temperature conditions might have been randomly chosen or not based on a recognized industry standard. Whereas, the present study focuses on the use of ISO 834 standard guidelines for heating the concrete samples. In contrast to the past studies, the current study is designed to follow the ISO 834 standard guidelines. ISO 834 is an internationally recognized standard that provides guidelines for simulating fire exposure on building elements, including concrete, in a controlled and consistent manner. This standard specifies a heating curve that represents the time-temperature relationship of a real building fire.

In this case, the concrete specimens were exposed to real-time fire exposure as set by ISO 834. The temperature conditions applied to the concrete specimens in the study closely mimic the heat that would be experienced by real building elements during a fire, following a specific time-temperature profile.

Utilization of Indian Standard for Alkali activated concrete: This research paper marks a pioneering effort by utilizing the guidelines specified in this Standard; it is the comprehensive code providing guidelines for development of the alkali-activated concrete.

Optimized Geopolymer Formulation: The study focuses on optimizing the geopolymer concrete mix design as per Indian Standard, considering locally available materials and environmental influencing factors. This contributes to the practicality and sustainability of geopolymer concrete application in various regions.

Thermal Resistance Assessment: Geopolymer concrete’s performance under extreme temperature conditions is a critical factor for its widespread application. The paper rigorously evaluates the concrete’s behavior when subjected to elevated temperatures following the ISO 834 standard guidelines. This evaluation provides valuable insights into its fire resistance and suitability for applications in fire-prone areas.

Enhanced Infrastructure Resilience: The research outcomes have the potential to significantly enhance the resilience of critical infrastructure, such as bridges, tunnels, and high-rise buildings, by offering a sustainable and fire-resistant alternative to traditional concrete.

## 2. Materials and Methods

### 2.1. Materials

#### 2.1.1. Binder

Low calcium Fly ash (FA) obtained from coal fired thermal power plant, Mettur, India. Ground granulated blast furnace slag (GGBS) obtained from JSW steel plant, Salem, India. FA and GGBS was employed as the binder material for the production of concrete. Table 1, illustrates the oxide composition of FA and GGBS. The specific gravity of the FA and GGBS were found to be 2.25 and 2.96, respectively. Specific surface area of FA and GGBS was found to be 440 m^2^/kg and 510 m^2^/kg.

#### 2.1.2. Filler

**Figure 1:**
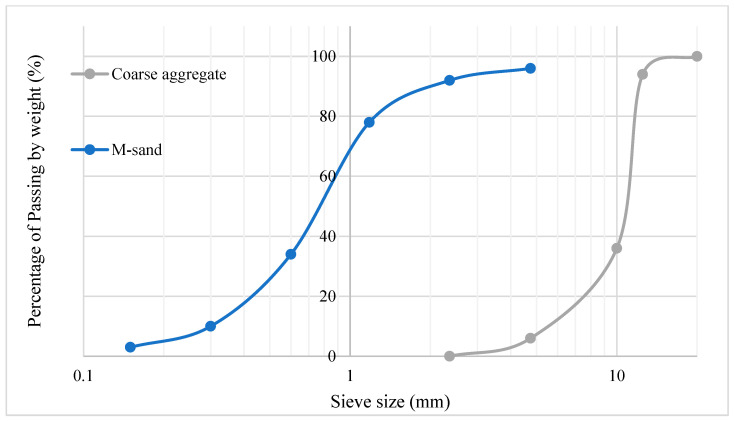
Particle Size distribution of fine and coarse aggregate.

Due to scarcity of river sand, the manufactured sand (M-sand) was employed as the fine aggregate in the construction industry. The crushed granite stones were employed as the coarse aggregate for the production of concrete. The M-sand of size 4.75 mm and below were employed, whereas coarse aggregate of size 12.5 and 10 mm, in the ratio of 60:40 was employed for the concrete production. Particle size distribution of fine and coarse aggregate was depicted in Figure 1. Coarse and Fine aggregates conform to the requirements of IS 383 [25], were employed for the preparation of concrete. The specific gravity of coarse aggregate is 2.73 and the specific gravity of fine aggregate is 2.72. Whereas the density of coarse aggregate and fine aggregate was found to be 1540 kg/m^3^ and 1821 kg/m^3^.

#### 2.1.3. Alkaline Activator

Sodium hydroxide (NaOH) conforming to IS 252 [26], and Sodium silicate (Na_2_SiO_3_) (alkaline grade) conforming to IS 381 [27] were employed as the alkaline activator for the production of GPC. The specific gravity of the NaOH and Na_2_SiO_3_ employed in the present investigation is 1.29 and 1.55. Table 2, illustrates the concrete with different grades and varying W/B ratio employed in the present investigation. Further Table 3, illustrates the mix design adopted in the present study.

### 2.2. Methods

#### 2.2.1. Casting & Curing

Initially, the binder and the filler materials are mixed in dry state, then followed by the addition of activator solution. The mixing was continued until the uniform consistency was achieved. After achieving the uniform consistency, the freshly prepared mix was checked to assess its physical properties. After which the mix was poured into the mold of required size to assess its mechanical properties. The mix cast was allowed to set for a period of 24 h, beyond which the concrete samples were demolded and allowed in ambient or in oven conditions until the corresponding day of testing.

The choice of curing method for GPC largely depends on the raw materials and mix composition used. Various methods, such as thermal curing, water curing, and curing with compounds, can be employed to achieve the desired concrete properties. However, it is important to note that the optimal curing method may vary depending on the specific system being used. For instance, thermal curing may be more appropriate for low-calcium systems, while air or water curing may be more suitable for high-calcium systems. Additionally, it is crucial to take measures to prevent the leaching of alkali activators during the curing process.

#### 2.2.2. Testing

As per IS 516 [28], CS of the concrete mix was found by testing the cube specimen of size 150 mm × 150 mm × 150 mm. After the completion of required days of curing the specimens were tested in the compression testing machine of capacity 2000 kN.

The sorptivity of a GPC mix, which refers to the process of water molecules moving upwards from the base to the top of the concrete specimens through capillary action within a specific time, was determined according to the ASTM C 1585 [29] standard. To conduct the sorptivity test, a disk-shaped concrete specimen with a diameter of 75 mm was utilized. The side surface of the specimen was coated with a layer of asphalt, and tape was applied to render the side surfaces impermeable. The geopolymer specimens were then immersed in a water tub, with the water level maintained approximately 1 cm above the base of the specimens. The initial weight of the concrete specimens was recorded under ambient conditions. At specific time intervals of 10, 20, 30, 40, 50, 60 min, 6 h, 12 h, and 24 h, the geopolymer specimens were removed from the water tub. The rate of water absorption (WA) of the geopolymer specimens was calculated using Equation (1).

(1) $$I=\left(\frac{m_{t}}{a/\;d}\right)$$

where *I* denote the absorption, m_t_ is the change in mass in grams, a is the area of the specimen, and d is the density of water.

#### 2.2.3. Heating Program

The purpose of the experiment was to evaluate the residual strength of the GPC that had been developed. To achieve this, the concrete samples were subjected to varying levels of heat using an electric furnace with a maximum temperature capacity of 1200 °C. The heating procedure adhered to the ISO 834 [30] standard guidelines, ensuring consistency and reliability. The concrete samples underwent four distinct heating scenarios: heating to 821 °C for 30 min, 925 °C for 60 min, 986 °C for 90 min, and 1029 °C for 120 min. Once the target temperature was reached, the furnace automatically turned off, allowing the samples to cool naturally to room temperature. Subsequently, the cooled samples were carefully examined to quantify the extent of mass loss and determine the remaining CS. To ensure precision and accuracy, a minimum of three samples were tested for each grade during the evaluation process.

#### 2.2.4. Crack Width Measurement

After heating the concrete specimens in the computerized electric furnace at the target temperature, the specimens were cooled under room temperature. After which the thermal cracks developed on the surface of the concrete specimen were measured using “Elcometer”. Elcometer provides a low-cost microscope for determining the width of a crack in concrete or other building materials.

#### 2.2.5. Scanning Electron Microscope

The Scanning Electron Microscope (SEM) was performed using a high range of Phenom ProX desktop SEM. The resolution of the SEM was not less than 8 nm with an increased depth field and an accelerating voltage of 15 kV. The samples are procured from 28-d aged CS tested specimens to examine the polymerization rate and sufficiency of geopolymer gel in the matrix.

## 3. Results

### 3.1. Compressive Strength (CS)

The W/B ratio is a critical factor that can affect the CS of GPC [8]. In general, as the water-to-binder ratio decreases, the CS of the GPC increases; this is because a lower W/B ratio leads to a stronger matrix with reduced porosity [9]. However, a very low W/B ratio can also have a negative impact on the CS [15]. If the mixture is too dry, it can be difficult to achieve proper mixing and compaction, which can result in a weaker concrete with lower strength [31]. Therefore, an optimum water-to-binder ratio should be selected based on the specific geopolymer mixture and curing conditions. The W/B ratio should be optimized to achieve a balance between workability and strength.

**Figure 2:**
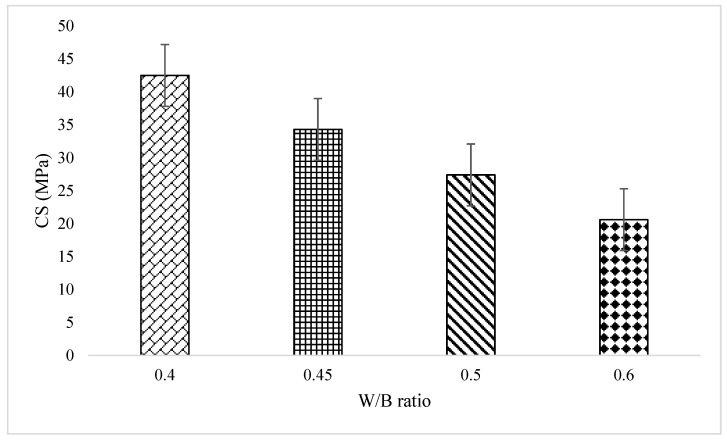
Relation between CS and W/B ratio.

Based on the experimental investigation, it is found that an increase in W/B ratio decreases the CS of the concrete. The mix with 0.6 W/B ratio, possess the lowest strength of 20.6 MPa, and the mix with low W/B ratio of 0.4 attained a CS of 42.5 MPa in accordance with IS 516 [28]. The strength vs. W/B ratio of GPC mixes were depicted in Figure 2. Table 4, describes the statistical data of all the samples. The higher CS of the geopolymer mix with a lower W/B ratio can be attributed to the fact that a lower amount of water leads to a more compact and denser concrete matrix, which enhances its strength. When the W/B ratio is reduced, the amount of water available for the reaction decreases, resulting in a more extensive reaction between the geopolymer binder and the available silica and alumina sources in the mix. This reaction produces a more stable and denser structure, which leads to the higher CS of the geopolymer mix.

It is worth noting that the CS of the geopolymer mix is highly influenced by various factors. Hence, to achieve the desired strength and performance, it is crucial to carefully select the mix design and optimize the various parameters involved in the geopolymer mix.

Further, the CS of the GPC was influenced by several factors, including:

Chemical composition [32]: The chemical composition of the geopolymer binder plays a crucial role in determining the CS of the concrete. The use of different types of precursors, activators, and additives can affect the chemical reaction and ultimately the strength of the geopolymer.

Curing conditions [33]: The curing conditions, such as temperature and humidity, can significantly impact the CS of GPC. Proper curing can enhance the development of the geopolymer matrix, resulting in increased strength.

Aggregate type and size [34]: The type and size of the aggregate used in GPC can affect its strength. Aggregates with high strength and low absorption properties are preferred to ensure better bonding with the geopolymer matrix.

W/B ratio [35]: The W/B ratio is a critical factor that can affect the CS of GPC. A lower water-to-binder ratio can lead to a stronger matrix with reduced porosity.

Age [31]: The CS of GPC increases with age. The curing time should be optimized to allow sufficient time for the geopolymerization reaction to occur and for the concrete to gain strength.

Testing methods [36]: The CS of GPC can be influenced by the testing method used. Standardized testing procedures, such as IS 516 [28], should be followed to ensure accurate and reliable results.

### 3.2. Sorptivity

The sorptivity test is a common method for evaluating the permeability and durability of concrete [37]. It measures the rate of water absorption into the concrete surface under a given pressure. The sorptivity of GPC can be influenced by several factors, including the chemical composition, curing conditions, and age of the concrete [38]. In GPC, the sorptivity is often lower than that of traditional Portland cement concrete [39]. This is because geopolymerization produces a denser microstructure with reduced porosity, which limits the ingress of water and other harmful substances [40]. The sorptivity test can be used as a tool to assess the durability and resistance of GPC to various environmental conditions, such as freeze-thaw cycles, chemical attack, and moisture exposure [41]. A lower sorptivity indicates better resistance to these factors and, hence, longer service life [42]. Therefore, optimizing the sorptivity of GPC is essential for improving its overall performance and durability.

**Figure 3:**
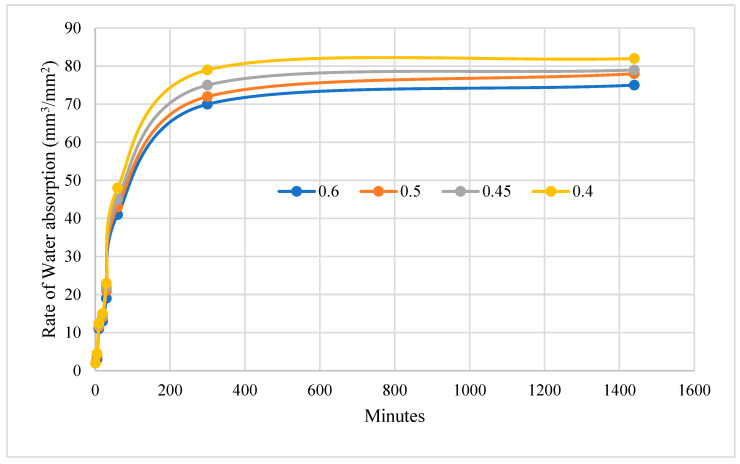
Sorptivity test results of GPC with varying W/B ratio.

The sorptivity of GPC can be further reduced by optimizing the curing conditions, such as temperature and humidity, to enhance the development of the geopolymer matrix [43]. The sorptivity of GPC can also vary depending on the type and proportion of the precursors and activators used [44]. For example, the use of fly ash or slag as precursors can result in a lower sorptivity compared to other materials. From the experimental investigation, it is found that the mix with lower W/B ratio possess a maximum absorption than the mix with higher W/B ratio as shown in Figure 3. This is because, as the W/B ratio decreases, the amount of water available for mixing with the binder decreases, resulting in a lower workability of the mix [45]. This, in turn, leads to an increased number of voids or pores in the final GPC structure, which results in a higher rate of water absorption.

In addition, lower W/B ratio GPC mixes tend to have a denser microstructure due to the reduction in porosity, which makes it more difficult for water to penetrate into the concrete matrix. However, the higher number of voids or pores present in lower W/B ratio mixes can offset this effect and lead to a higher rate of absorption. It is worth noting that the rate of absorption of GPC is also influenced by other factors such as the type and amount of activators, curing conditions, and the nature of the aggregate used [46]. Therefore, the W/B ratio should be optimized along with other factors to achieve the desired properties of the GPC.

### 3.3. Mass Loss

**Figure 4:**
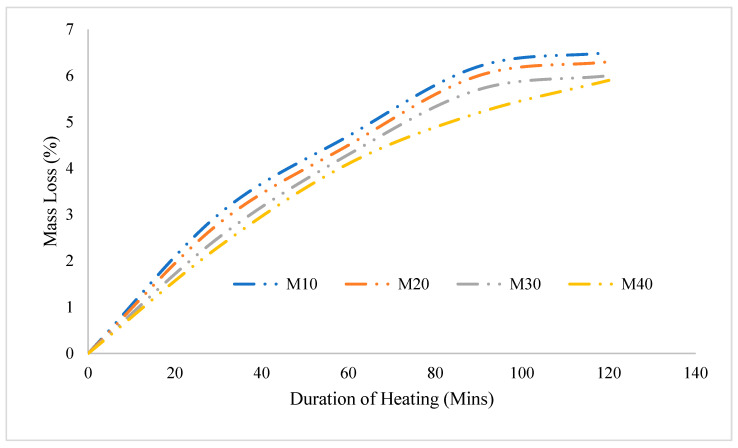
Mass Loss of concrete after temperature exposure.

The mass loss (ML) of all the concrete specimens was evaluated after they were exposed to varying temperatures, and the results are shown in Figure 4. To analyses the ML, the concrete samples were weighed before and after being subjected to temperature exposure. By comparing the weights, the extent of moisture loss can be determined. Figure 4 illustrates the relationship between the ML and the temperature exposure. It is evident from the graph that as the temperature exposure increases, the ML decreases. The mass loss of the M10 mix, after being exposed to a temperature for 30 min, was determined to be 3%. However, when the exposure time was extended to 120 min, the mass loss increased to 6.5%. Similarly, for the M20 grade concrete, the moisture loss (ML) was measured to be 0.8% and 6.3% after subjecting it to 30 and 120 min of exposure, respectively. Moving on to the M30 grade concrete, the ML was found to be 2.5% after 30 min of exposure, it is then increased to 6% after 120 min of exposure. For the M40 grade concrete, the observed mass losses were 2.3% and 5.9% for 30 and 120 min of exposure, respectively.

These results indicate that as the grade of the concrete increases (from M10 to M40), the amount of moisture loss also tends to increase. Moreover, the longer duration the specimens are exposed to elevated temperatures, the greater the mass loss becomes. These findings suggest that the M10 mix exhibits relatively lower moisture loss compared to the higher-grade concretes (M20, M30, and M40) under the same temperature exposure conditions. The experimental study revealed that concrete with a lower water-to-binder (W/B) ratio exhibited superior performance compared to concrete with a higher W/B ratio when subjected to elevated temperatures. This observation can be attributed to the generation of high internal pressure within the concrete having a high W/B ratio during temperature exposure. The high W/B ratio in concrete means that there is a greater amount of water present relative to the amount of cementitious binder. When this type of concrete is exposed to elevated temperatures, the water within it undergoes vaporization and turns into steam. The conversion of water into steam generates significant internal pressure within the concrete mass.

The accumulation of high internal pressure within the concrete with a high W/B ratio leads to several detrimental effects [47]. One major consequence is the weakening of the interfacial transition zone between the binder and the filler materials in the concrete [48]. This interfacial transition zone is crucial for transmitting stress and ensuring the overall strength and integrity of the concrete [49]. As the internal pressure increases, it exerts stress on the interfacial transition zone, causing it to become compromised and less effective in transferring loads. This compromised zone can result in a reduction in the strength of the concrete and contribute to mass loss [38]. In contrast, concrete with a lower W/B ratio has a reduced water content, resulting in a smaller amount of internal pressure generated during temperature exposure. As a result, the interfacial transition zone between the binder and filler materials remains more intact, maintaining better strength and minimizing mass loss [50]. Based on these findings, it can be concluded that utilizing concrete with a lower W/B ratio is advantageous in terms of maintaining structural integrity and minimizing strength and mass loss when exposed to elevated temperatures [51].

### 3.4. Crack Pattern

The examination of crack intensity at the concrete surface is a widely utilized method to assess the deterioration of concrete after exposure to elevated temperatures [52]. This practice is commonly adopted as a reliable means to evaluate the extent of damage caused by temperature effects. To determine the cracks’ characteristics on the concrete surface, a commonly used tool called an elcometer or crack measurement microscope was employed. This device possesses the capability to measure crack widths up to a maximum of 1.8 mm, allowing for precise and accurate crack assessments. In the specific study or research being referenced, the elcometer was utilized to measure the crack widths resulting from temperature exposure. By employing this tool, researchers were able to obtain quantitative measurements of crack widths at the concrete surface, providing valuable data for assessing the extent of damage. The elcometer or crack measurement microscope aids in accurately capturing the dimensions of cracks, enabling researchers to determine the intensity of cracking and evaluate the severity of concrete deterioration. This information is essential for understanding the performance and durability of the concrete when subjected to elevated temperatures.

**Figure 5:**
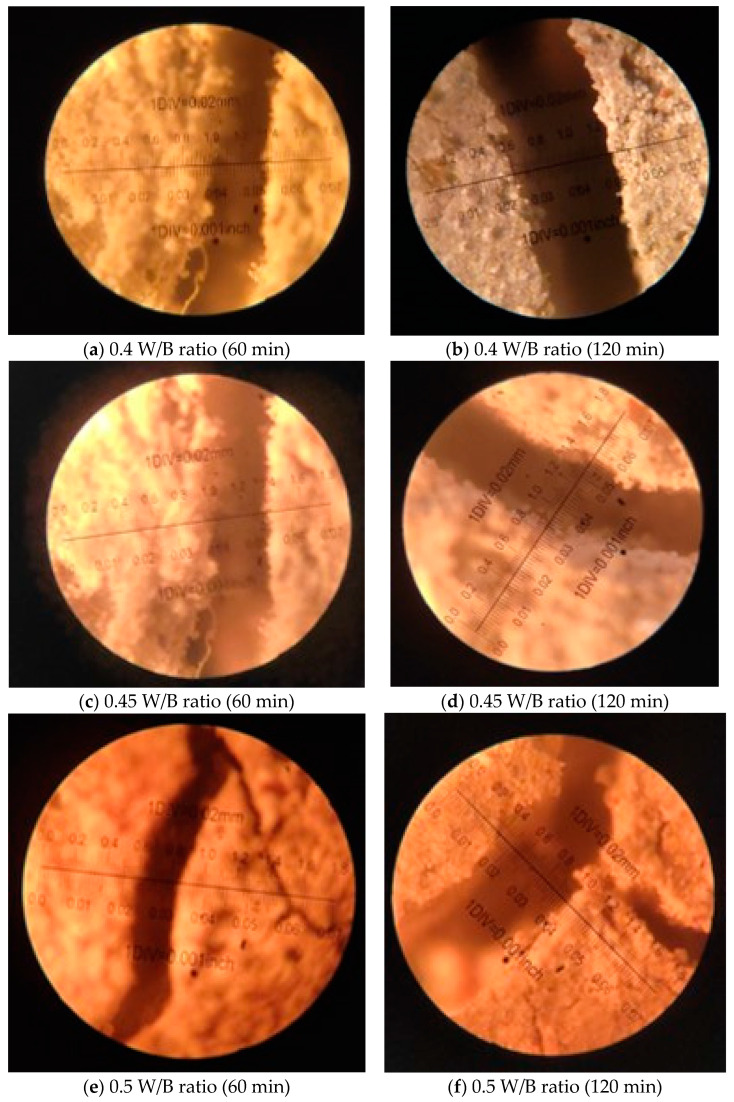

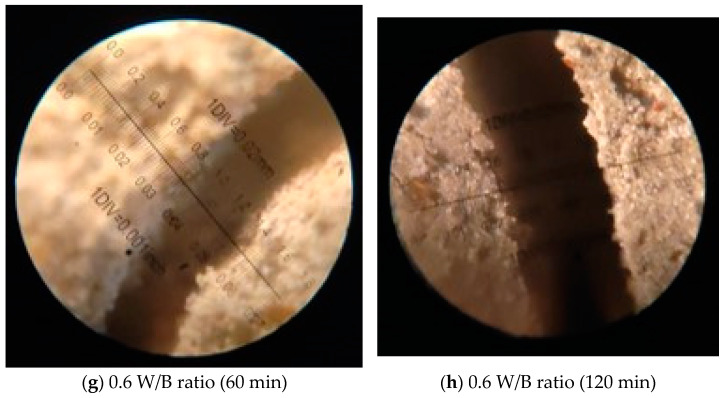
Crack width of GPC with varying W/B ratio.

By utilizing the elcometer to measure crack widths up to 1.8 mm, researchers can gather precise data, enabling quantitative analysis of crack patterns and intensities. This quantitative assessment offers insights into the damage mechanisms, helps to identify critical areas of deterioration, and informs decisions regarding potential repair strategies or modifications to enhance the concrete’s resistance to thermal stresses. Figure 5, provides a visual representation of the concrete surface after being exposed to elevated temperatures. In the study, all the concrete specimens, ranging from M10 to M40 grades, were subjected to an initial heating temperature of 821 °C for a duration of 30 min. Upon this initial exposure, cracking was observed to initiate in all the concrete specimens. The appearance of cracks indicated that the elevated temperature had induced thermal stresses in the concrete, leading to the formation of visible fractures on the surface. However, the intensity of cracking became more pronounced when the specimens were further exposed to a higher temperature of 1029 °C for a longer duration of 120 min. This extended exposure time to higher temperatures intensified the thermal stresses acting on the concrete, resulting in more extensive and severe cracking.

The observed cracks in the concrete specimens were a direct consequence of the thermal expansion and contraction experienced during the heating and cooling cycles. As the temperature increased, the concrete expanded, and when cooled, it contracted. These thermal movements generated internal stresses that exceeded the tensile strength of the concrete, causing to the development of crack. The increased cracking intensity witnessed at the higher temperature and longer exposure duration highlights the vulnerability of concrete to thermal stresses [53]. It underscores the importance of understanding and addressing the potential impact of elevated temperatures on concrete structures. The findings from Figure 5, emphasize the need for appropriate design considerations and material selection to mitigate the adverse effects of thermal stresses. The crack widths of different concrete grades were measured to assess the extent of cracking.

Figure 5a,b shows the crack width details of blended concrete with 0.4 W/B ratio (M40), the crack widths were measured in the range of 0.6 mm to 0.8 mm. Figure 5c,d depicts the details of concrete with 0.45 W/B ratio (M30), the crack widths were found to be between 0.4 and 0.6 mm after exposing to 60 and 120 min of heating. In the case of 0.5 W/B ratio (M20), the crack width was in the range of 0.3 mm and 0.6 mm. In the case of 0.6 W/B ratio (M10) blended concrete, the observed crack widths ranged between 0.4 mm and 0.8 mm. This indicates that the cracks on the surface of the 0.6 W/B ratio blended concrete were relatively narrow, falling within the measured range as shown in Figure 5g,h. These measurements indicate that the cracks observed on the surface of the 0.4 W/B ratio blended concrete were slightly wider compared to the 0.6 W/B ratio blended concrete. The crack width of concrete is a significant parameter to consider, as it provides insights into the degree of cracking and the potential impact on the structural integrity. Narrower cracks indicate a relatively lower level of cracking, whereas wider cracks suggest a greater extent of damage and potential compromise of the concrete’s performance. The differences in crack widths between the 0.6 W/B ratio blended concrete and 0.4 W/B ratio blended concretes may be attributed to variations in the composition, strength, and other properties of the concrete mixtures. Factors such as water-to-binder ratio, aggregate characteristics, and curing conditions can influence the crack width formation [54].

### 3.5. Residual CS

**Figure 6:**
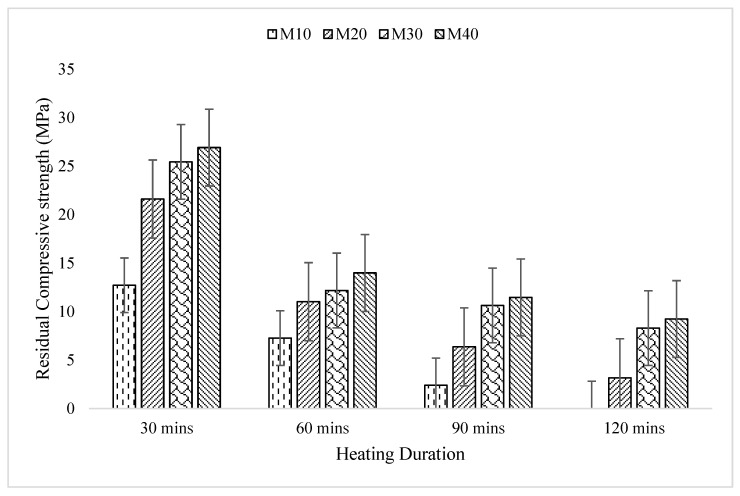
Residual strength of GPC after subjecting to elevated temperature of 821 °C (30 min), 925 °C (60 min), 986 °C (90 min) and 1029 °C (120 min).

The experimental investigation focused on evaluating the residual strength of GPC samples when exposed to varying temperatures. Figure 6, presented in the study illustrates the findings obtained from this investigation. Further Table 5 illustrates the statistical data of all the concrete samples. The objective was to assess, how the compressive strength (CS) of the GPC samples affected by different temperature exposures. The results revealed a consistent trend across all concrete grades, indicating a decrease in CS as the temperature exposure increased. The decrease in CS with increasing temperature exposure suggests that the elevated temperatures had a detrimental effect on the GPC. The heat exposure caused changes in the material properties, resulting in a reduction in its ability to withstand compressive forces [55]. Various factors contribute to the reduction in CS of GPC under elevated temperature conditions [56]. These may include the breakdown of the chemical bonds within the geopolymer matrix, thermal expansion and contraction, microstructural changes, and the generation of internal stresses [57].

The experimental investigation specifically analyzed the impact of temperature exposure on the CS of GPC, with a focus on the M40 and M10 concrete grades. The results, as shown in the study, provide valuable insights into the extent of strength loss experienced by these concrete grades under different exposure durations. For the M40 grade concrete, it was observed that after 30 min of temperature exposure, there was a substantial decrease in CS of about 37%. However, the strength loss significantly escalated to 77% after an extended exposure duration of 120 min. These findings demonstrate a substantial and progressive weakening of the concrete’s CS, as the temperature exposure duration increased. Similarly, the M10 grade concrete also exhibited a notable decrease in CS. After 30 min of temperature exposure, the strength loss was measured at 38%. However, the situation worsened when subjected to 120 min of exposure, resulting in a complete loss of CS (100%).

These findings highlight the significant negative impact of temperature exposure on the CS of GPC. The results indicate that both the M40 and M10 concrete grades experienced substantial strength reductions, which can be attributed to various factors such as thermal expansion, chemical reactions, and the breakdown of the material’s internal structure. Through the conducted trials and experiments, it was observed that concrete with a lower water-to-binder (W/B) ratio exhibited better performance compared to concrete with a higher W/B ratio. The W/B ratio is a crucial parameter in concrete mix design, representing the ratio of water content to the binder materials (such as cement) used in the mixture. A lower W/B ratio signifies a lower amount of water relative to the amount of binder, resulting in a denser and more compact concrete matrix. The findings from the trials indicated that concrete with a lower W/B ratio demonstrated improved performance in several aspects. One of the key advantages was enhanced strength and durability. The lower water content resulted in a higher concentration of binder materials, promoting better interfacial bonding and overall strength of the concrete [58]. Additionally, the reduced water content contributed to a denser microstructure, minimizing the potential for moisture ingress, which can lead to deterioration and damage over time [59].

Furthermore, concrete with a lower W/B ratio exhibited improved resistance to various types of deterioration mechanisms, including cracking, shrinkage, and chemical attack. The reduced water content helped to mitigate the potential for excessive shrinkage and cracking, as well as limiting the availability of water for chemical reactions that could degrade the concrete. In contrast, concrete with a higher W/B ratio demonstrated comparatively inferior performance. The increased water content in these mixtures resulted in a higher porosity and weaker interfacial bond strength [55]. This porosity provided pathways for moisture ingress, promoting the potential for freeze-thaw damage, alkali-silica reaction, and other forms of deterioration [56]. The observed differences in concrete performance between lower and higher W/B ratios underline the significance of appropriate mix design considerations. The findings from the study underscore the severe and detrimental effects of temperature exposure on the CS of GPC. The results clearly demonstrate that as the duration of exposure to elevated temperatures increases, the CS of GPC experiences a significant reduction.

These findings highlight the vulnerability of GPC to thermal stresses. When exposed to high temperatures, GPC undergoes various physical and chemical changes that negatively impact its structural integrity and strength [60]. The thermal stress induced by elevated temperatures can lead to the breakdown of the geopolymer matrix, causing a loss of intermolecular bonds and weakening the overall structure of the concrete [61]. The study’s results emphasize the need for implementing protective measures to mitigate the detrimental effects of temperature exposure on GPC [62]. Protective measures may include the use of insulation materials, fire-resistant coatings, or implementing cooling systems to minimize the temperature rise of the concrete during exposure. Additionally, incorporating additives or modifying the composition of the geopolymer mixture can enhance its resistance to high temperatures. These additives can help in reducing the thermal expansion and contraction of the concrete, improving its ability to withstand thermal stresses and preserving its CS. Understanding the vulnerability of GPC to temperature exposure is crucial for designing and constructing structures that can withstand fire incidents or other high-temperature environments [63]. By implementing appropriate protective measures and modifying the concrete mixture, researchers can enhance the resilience of GPC, enabling it to maintain its structural integrity even under severe thermal conditions.

### 3.6. Microstructural Investigation

**Figure 7:**
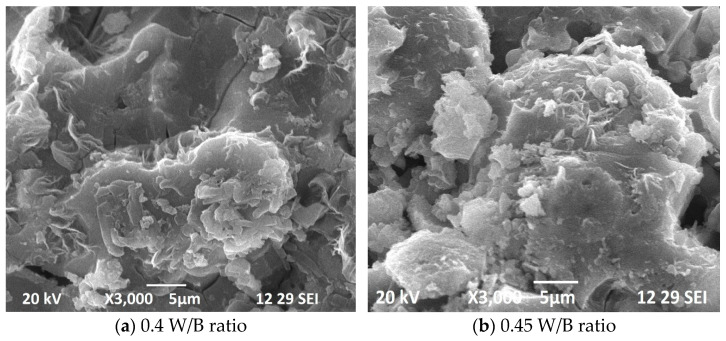

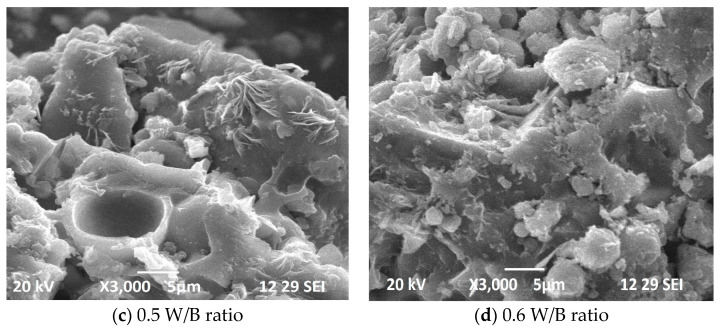
SEM image of GPC with varying W/B ratio.

Scanning Electron Microscopy (SEM) can be used to investigate the microstructure of GPC with varying water-to-binder (W/B) ratios. SEM images can provide detailed information on the morphology, and distribution of the phases present in the concrete matrix. When the W/B ratio is decreased in GPC, the microstructure becomes more compact and less porous. This is due to the formation of a denser geopolymer matrix, which reduces the amount of water that can penetrate into the concrete. SEM images of GPC with lower W/B ratios typically show a smoother and more homogenous surface, with fewer voids and pores compared to concrete mixes with higher W/B ratios as shown in Figure 7. The geopolymer matrix is also more continuous and better distributed, resulting in a more uniform microstructure. On the other hand, GPC mixes with higher W/B ratios tend to have a more porous and irregular microstructure. SEM images of these mixes typically show more voids and cracks, and a less continuous geopolymer matrix. This is due to the excess water present in the mix, which can lead to the formation of more pores and voids during the curing process. Overall, SEM investigation of GPC with varying W/B ratios can provide insights into the microstructure and porosity of the concrete matrix, which can help to optimize the mixture design and improve the performance of the concrete.

**Figure 8:**
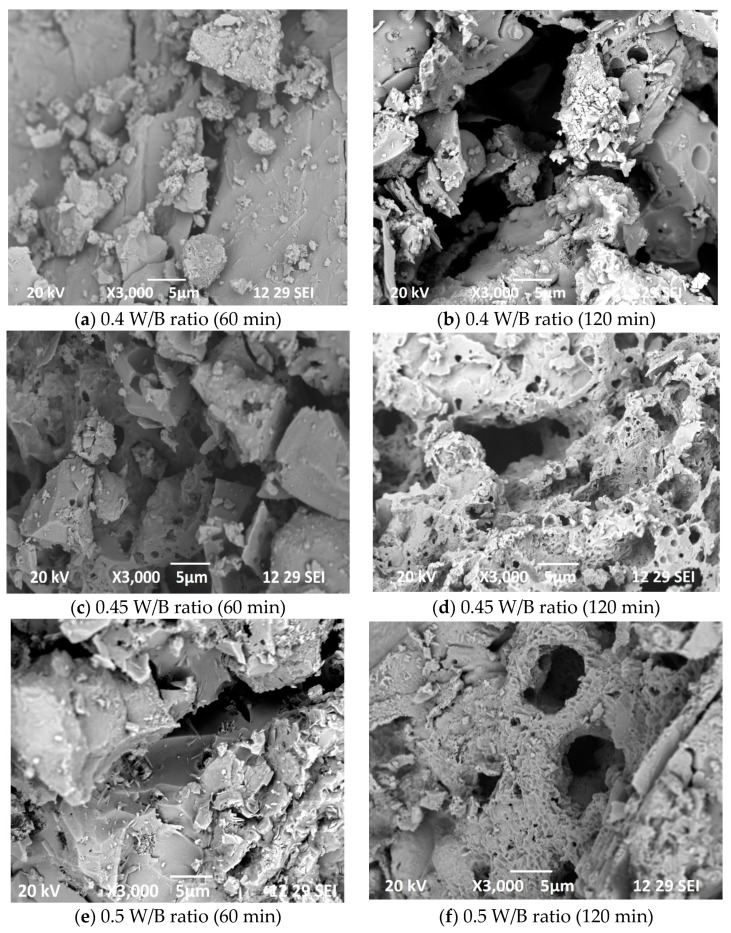

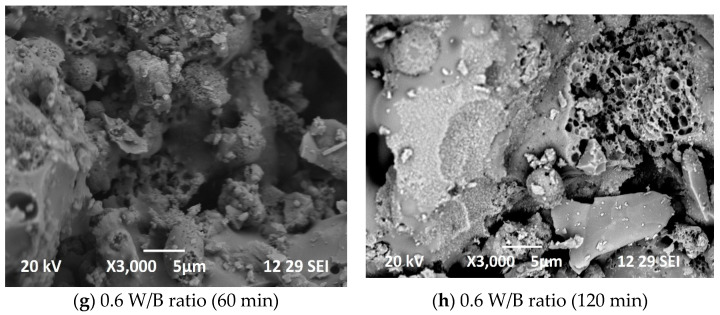
SEM images of concrete specimens after exposing to 60 and 120 min of exposure.

In Figure 8, the internal morphology of concrete specimens subjected to elevated temperature is depicted. The figure illustrates a noticeable alteration in the internal structure of the concrete samples before and after being exposed to elevated temperatures. The specific details of the observed changes are not mentioned in the given statement. However, the figure likely provides visual evidence of the modifications that occurred within the concrete’s internal composition due to the influence of temperature exposure. The SEM images revealed that an increase in temperature exposure led to several observable effects on the concrete’s morphology. Firstly, the exposure to higher temperatures resulted in an increase in the occurrence and size of cracks within the concrete structure. This suggests that elevated temperatures contribute to the development and propagation of cracks, which can potentially weaken the material. Secondly, the density of the concrete’s morphology was found to decrease with increasing temperature exposure. This implies that the internal structure of the concrete became less compact and more porous as the temperature rose. The reduction in density indicates that the concrete underwent changes in its composition, with the formation of voids or gaps within its matrix. Specifically, when the concrete specimens were subjected to a heating temperature of 925 °C, the SEM images exhibited a distinct porous structure. This indicates that the concrete experienced significant degradation and structural changes at this high temperature, resulting in the creation of interconnected pores or voids throughout the material. As the temperature exposure continues to rise, the deterioration of the concrete becomes more severe, ultimately leading to a significant loss of strength. This suggests that the adverse effects of temperature on concrete properties intensify with higher temperatures.

Upon subjecting the concrete specimens to temperatures of 925 °C and 1029 °C, it was observed that all concrete grades exhibited a similar behavior. This implies that the temperature-induced changes in the concrete were consistent across different grades of concrete. The cracks became more prominent and extensive, indicating a further degradation of the material. Additionally, the concrete exhibited an increased porosity, implying a higher number of interconnected voids or pores within its structure. This increased porosity further compromised the integrity and strength of the concrete. Figure 8a,b, shows the internal morphology of M40 grade concrete specimens after exposing to elevated temperature of 925 °C and 1029 °C. Figure 8c,d depicts the internal structure of M30 grade concrete specimens. Figure 8e,f shows the internal structure of M20 grade concrete specimens and Figure 8g,h, shows the internal morphology of M10 grade concrete specimens after exposing to 925 °C and 1029 °C. Based on the microstructure findings it was evident that the concrete specimens after exposing to 925 °C shows lower degradation when compared to the concrete specimens after exposing to 1029 °C. This shows that increase in the heating temperature increases the concrete degradation, and thus leads to strength degradation. Specimens exposing to 1029 °C, shows higher void formation. Due to increased number of voids in the concrete specimens, the bond between the binder and filler materials may be affected, this was confirmed in the residual compressive strength of concrete after subjecting to temperature exposure.

## 4. Discussion of the Results with Other Published Data

A number of researchers have done similar experimental studies to the work presented herein and the most relevant of these are summarized in Table 6. From the data presented in these works, following observations are deduced: The strength of concrete is reduced when it is exposed to high temperatures and exposure to elevated temperatures can also reduce the durability of concrete.

### 4.1. Following Are the Assumptions and Limitations Made during the Experimental and Analytical Investigation

- The comparison between the performance of binary blended mix is based on a specific mix proportion of materials used for blending, and the results may vary for other combinations.
- The decline in CS of concrete after temperature exposure is found for all samples tested, but there may be variations due to factors such as mix composition, curing conditions, and testing methods.
- The results are based on a limited proportion of samples, and the influence of various parameters on the performance of concrete needs further investigation.

### 4.2. Future Research Directions and Recommendations Based on the Findings of This Study Could Include

- Conducting more extensive experiments with a larger number of samples to establish a more reliable database to improve the statistical significance of the results through software simulation and modelling.
- Investigation on the influence of various parameters, such as precursor type, alkaline activator, activator concentration, reinforcement ratio, intensity and duration of heating, on the performance of concrete.
- Exploring the potential usage of other Supplementary cementitious materials, such as rice husk ash, bagasse ash and silica fume, into the mix to study their effect on the strength and durability of concrete.
- Investigation on the long-term behavior of concrete under different environmental conditions, such as freeze-thaw cycles and chloride ingress.
- Conducting a comparative life cycle assessment (LCA) of concrete with other sustainable construction materials to gain a better understanding of their environmental impact to identify potential areas for improvement.
- Investigating the potential of using recycled materials in the production of concrete to enhance their sustainability credentials.

## 5. Conclusions

In this study, a straightforward and effective mix design method is proposed for the development of geopolymer concrete (GPC) using fly ash and ground granulated blast furnace slag (GGBS) as key components. Unlike traditional concrete mix design, which focuses on factors like fine and coarse aggregate quantities, alkaline activator solution, and binder proportions, the proposed approach incorporates the influence of specific gravity of raw materials in the mix design.

By adopting this innovative methodology, consistent outcomes are attainable despite variations in the physical attributes of raw materials. Notably, the research reveals that the fundamental principle of strength relative to W/B ratio, as outlined in the Indian standard, is equally relevant to GPC production.

Furthermore, proposed study establishes that GPC can achieve compressive strengths (CS) ranging from 10 MPa to 40 MPa after 28 days, even under ambient temperature conditions. Importantly, the results underscore that GPC exhibits superior CS when compared to conventional concrete with identical liquid content.

In terms of durability, concrete specimens featuring a W/B ratio of 0.6 exhibit mass losses within the range of 3% to 6.5%. In contrast, specimens with a lower W/B ratio of 0.4 experience slightly lower mass losses, ranging from 2.3% to 5.9%.

Analyzing strength degradation, it becomes evident that concrete with a W/B ratio of 0.4 demonstrates a reduction in strength from 36.81% to 77.09%. Conversely, concrete formulated with a W/B ratio of 0.6 displays a somewhat higher strength loss, ranging from 38.30% to 100%.

## Figures and Tables

**Table 1:** Oxide composition of FA and GGBS.

Oxides	FA (%)	GGBS (%)
SiO_2_	61.2	42.4
Al_2_O_3_	26.9	13.2
Fe_2_O_3_	6.21	1.12
Cao	1.91	41.2
MgO	0.29	1.3
Na_2_O	0.58	0.3
K_2_O	1.21	0.7
Ti	0.4	-
LOI	0.89	0.52

**Table 2:** Typical grades of concrete with varying W/B ratio.

**Sl.no**	**Target CS (MPa)**	**Minimum Binder Material Content (kg)**	**Maximum Total Water-Binder Material Ratio**
1	10	280	0.6
2	20	320	0.5
3	30	350	0.45
4	40	400	0.4

**Table 3:** Mix proportions.

Mix Type	FA	GGBS	Fine Aggregate	Coarse Aggregate	Na_2_SiO_3_	NaOH
	(kg/m^3^)					
M40	280	120	803	1156	51.84	34.56
M30	245	105	750	1080	48	32
M20	224	96	653	970	45.32	30.21
M10	196	84	450	650	43.44	28.96

**Table 4:** Compressive strength of the concrete samples.

Grades	Sample 1	Sample 2	Sample 3	Mean	SD	CoV (%)
M10	19.8	20.8	21.2	20.60	0.72	0.04
M20	27.4	26.05	28.9	27.45	1.43	0.05
M30	33.71	33.89	35.3	34.30	0.87	0.03
M40	42.06	43.1	42.65	42.60	0.52	0.01

Key: SD—Standard deviation; CoV—Co-efficient of variation.

**Table 5:** Residual compressive strength of the concrete samples after exposing to elevated temperature.

Grades	Duration	Sample 1	Sample 2	Sample 3	Mean	SD	CoV (%)
M10	30 min	13.21	12.14	12.98	12.78	0.56	0.04
M20		21.52	20.7	22.63	21.62	0.97	0.04
M30		24.98	25.06	26.3	25.45	0.74	0.03
M40		25.88	26.94	27.9	26.91	1.01	0.04
M10	60 min	6.5	7.2	8.1	7.27	0.80	0.11
M20		10.5	10.8	11.75	11.02	0.65	0.06
M30		10.89	12.45	13.2	12.18	1.18	0.10
M40		13.72	14.05	14.21	13.99	0.25	0.02
M10	90 min	1.98	2.65	2.57	2.40	0.37	0.15
M20		6.2	5.8	7.1	6.37	0.67	0.10
M30		10.5	10.2	11.2	10.63	0.51	0.05
M40		10.8	12.1	11.5	11.47	0.65	0.06
M10	120 min	-	-	-	-	-	-
M20		3.5	3.1	2.9	3.17	0.31	0.10
M30		6.89	8.8	9.2	8.30	1.23	0.15
M40		10.2	8.6	8.9	9.23	0.85	0.09

**Table 6:** Similar experimental data presented in the literature for the residual properties of various concrete composites following exposure to elevated temperature.

Ref.	Type of Concrete	Binder Medium	Temperature (°C)
[64]	GPC	Fly Ash	400, 600 and 800 °C
Increase in the activator to binder ratio from 0.5 to 0.7 increased the weight loss by an average of 1% at each of the temperatures of 400 °C, 600 °C and 800 °C, respectively. Increasing the activator ratio from 1.5 to 3.5 decreased weight loss on average by 0.4% at 400 °C, 0.9% at 600 °C and 0.7% at 800 °C. Increase in the activator ratio, compressive strength decreased from 13% to 31% for at 400 °C, from 45% to 53% at 600 °C, and around 80% at 800 °C			
[65]	Fiber reinforced GPC	Fly Ash and GGBS	200, 500 and 800 °C
The residual compressive strength of GPC at 200 was greater than its original strength. there was a strength enhancement of 11.1% which is ascribed to the secondary geopolymerization. After exposure to 500 and 800 °C, all the concrete specimens showed an obvious decline in compressive strength			
[66]	Lightweight geopolymer concrete	Fly Ash and GGBS	100 °C to 800 °C
Heating the concrete samples to 100 °C contributed to increasing the compressive strength of geopolymer and cement concrete. Raising the heating temperature to above 100 °C led to a loss in the compressive strength of the cement concrete and geopolymer. The behavior of the compressive strength of geopolymer concrete was compatible with cement concrete under the heating influence of the entire temperatures			
[67]	Ultra-high performance geopolymer concrete (UHPGC)	GGBS, Silica fume, waste glass (WG) and waste ceramic (WC)	200–800 °C
The residual strength of UHPGC containing WG varied from 98% to 97%, 59–63%, and 27–32% after being subjected to 300, 600, and 800 °C, respectively. While WC samples had residual strengths varying from 86% to 83%, 51–45%, and 24–18%, respectively.			
[68]	GPC	Metakaolin	200 °C, 400 °C, and 600 °C
Compressive strength was lost at a higher rate when temperature increased from ambient to 200 °C; however, the rate of reduction was lower when temperature increased from 200 °C to 400 °C and from 400 °C to 600 °C. Also, the residual compressive strength varied from 56% to 63%, 38% to 51%, and 28% to 34% after exposure to 200 °C, 400 °C, and 600 °C			
[69]	GPC	Fly ash, metakaolin, and slag	100 °C to 700 °C
GPC mixes revealed the strength losses after being subjected to temperatures between 200 and 700 °C due to the thermal incompatibility between geopolymer matrix and aggregates resulting in debonding and internal cracks.			
[70]	GPC	Fly ash and GGBS	100 °C to 800 °C
When exposed to elevated temperatures, the compressive strength of alkali activated fly ash (AAF) shows a sharp rise before 200 °C, and then largely maintains the strength gain effect from 200 to 800 °C with a 150.3% improvement recorded after 800 °C exposure			
Present	GPC	Fly ash and GGBS	821 °C, 925 °C, 986 °C and 1029 °C.
Most of the past studies deal with the concrete subjecting to random temperature exposure. This means that in previous studies, researchers subjected concrete samples to varying and unspecified temperature conditions, possibly simulating fire or heat exposure, but without adhering to a specific standardized guideline. The temperature conditions might have been randomly chosen or not based on a recognized industry standard. Whereas the present study focuses on the use of ISO 834 [30] standard guidelines for heating the concrete samples. In contrast to the past studies, the current study is designed to follow the ISO 834 [30] standard guidelines. ISO 834 [30] is an internationally recognized standard that provides guidelines for simulating fire exposure on building elements, including concrete, in a controlled and consistent manner. This standard specifies a heating curve that represents the time-temperature relationship of a real building fire. In this case, the concrete specimens were exposed to real-time fire exposure as set by ISO 834. The temperature conditions applied to the concrete specimens in the study closely mimic the heat that would be experienced by real building elements during a fire, following a specific time-temperature profile. The findings from this investigation underscore the vulnerability of geopolymer concrete, particularly in the M40 and M10 grades, to the adverse effects of temperature exposure. The study highlights a clear pattern of diminishing compressive strength with longer durations of elevated temperature exposure, focuses on the importance of considering these factors in the design and application of geopolymer concrete in scenarios where temperature fluctuations are anticipated.			

## Data Availability

Not applicable.
